# Supplementary material for: Well-defined double hysteresis loop in NaNbO3 antiferroelectrics
Source: Nat Commun. 2023 Mar 30;14:1776. doi: 10.1038/s41467-023-37469-x (PMC10063644; doi:10.1038/s41467-023-37469-x)
Supplement: Supplementary file 1 — Supplementary information [file 41467_2023_37469_MOESM1_ESM.pdf]

Supplementary Information

Well-defined double hysteresis loop in $NaNbO_3$ antiferroelectrics

Luo et al.

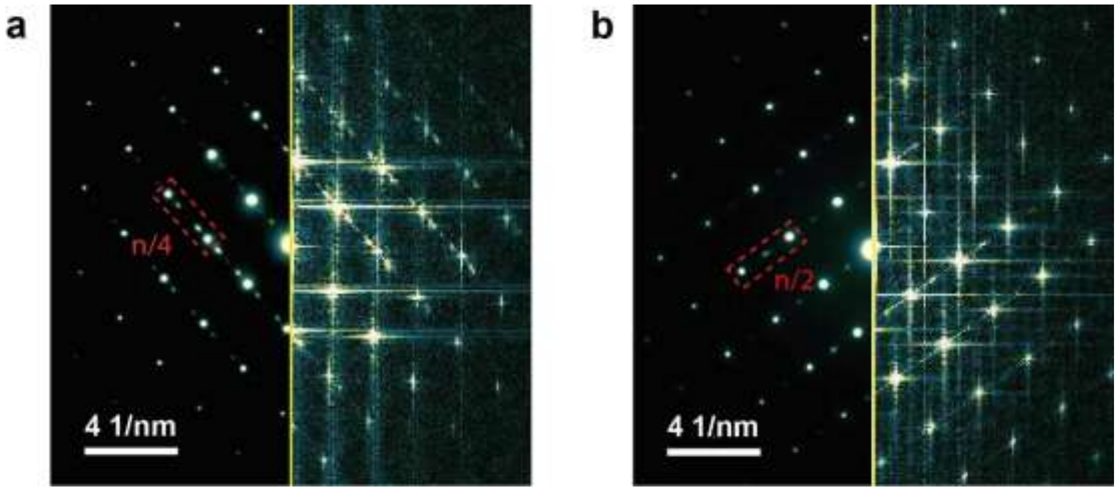

Supplementary Fig. 1 a) Selected area electron diffraction (SAED, left panel) and fast Fourier transform (FFT, right panel) of Fig. 1g showing n/4 modulations, identifying the P phase. b) SAED (left panel) and FFT (right panel) of Fig. 1h showing n/2 super spots, identifying the Q phase.

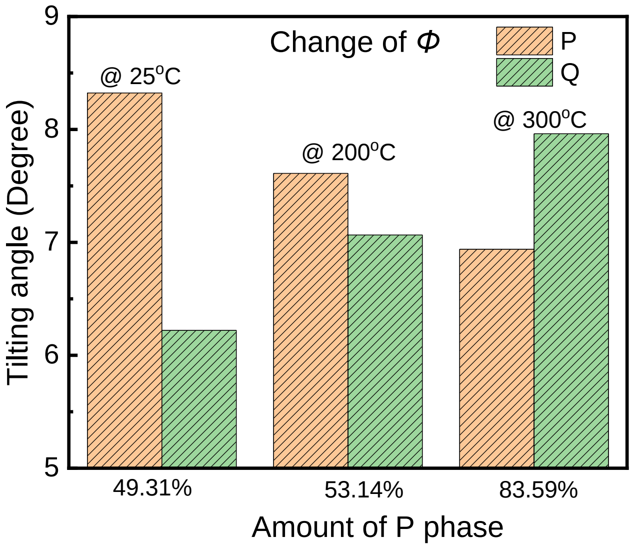

Supplementary Fig. 2 The [NbO_6] octahedral tilting angle Φ of P and Q phases as a function of P phase content for NN at different temperatures. The data are derived from the atomic coordinates presented in reference 1.

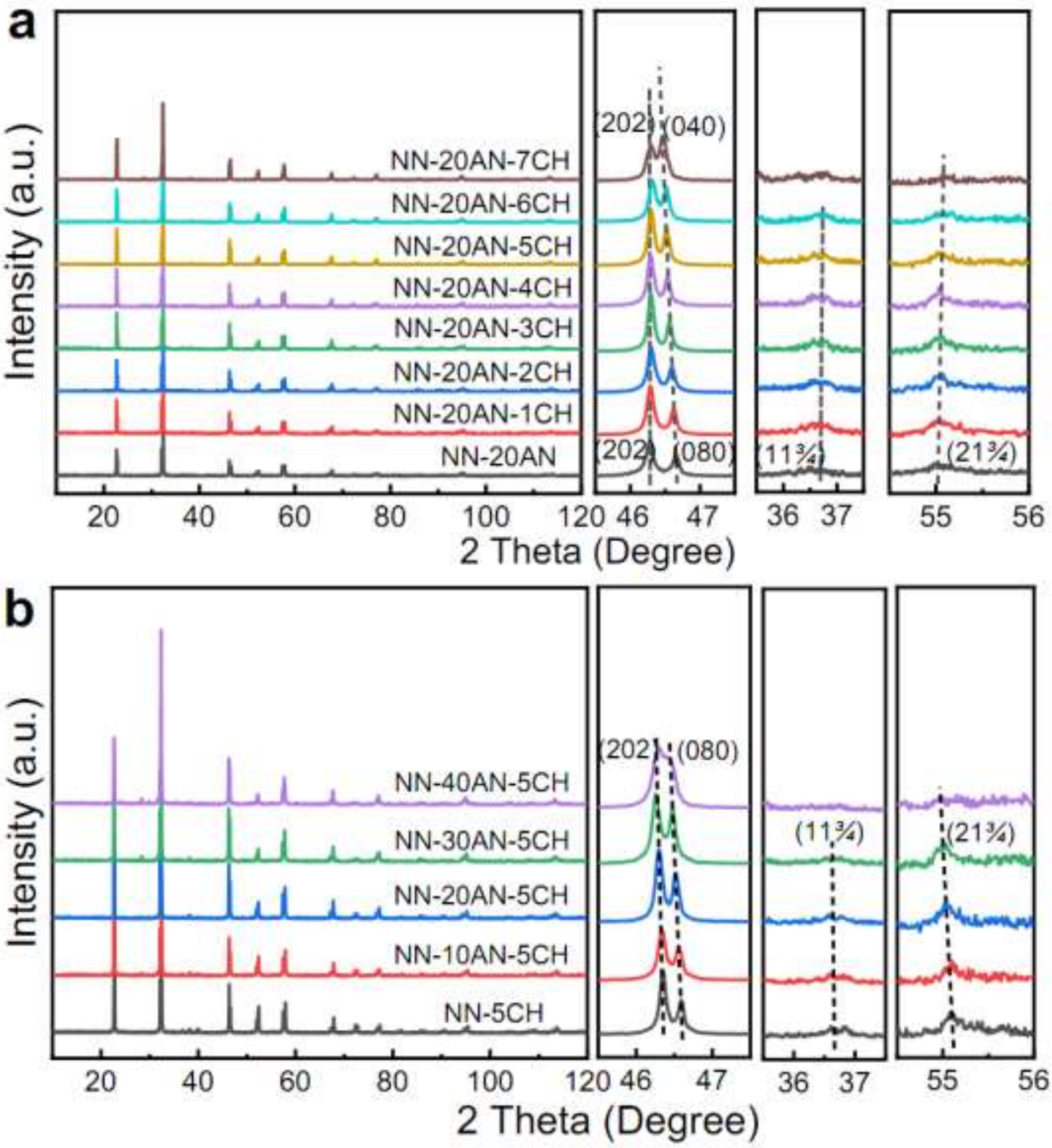

Supplementary Fig. 3 XRD patterns of a) NN-20AN-100*y*CH and b) NN-100*x*AN-5CH systems.

The NN-20AN exhibits typical orthorhombic perovskite structure with superlattice reflections of ($11^3/_4$) and ($21^3/_4$), indicating an AFE P feature[2]. A low level addition of CH in NN-20AN also keeps an AFE P structure. Once the amount of CH excesses 7mol%, another orthorhombic AFE R phase come to across, as indicated by the change of relative intensity of the (202)/(008) peaks around 46-47°.[3] The NN-5CH also exhibits AFE P structure, which keeps almost the same after the addition of AN component.

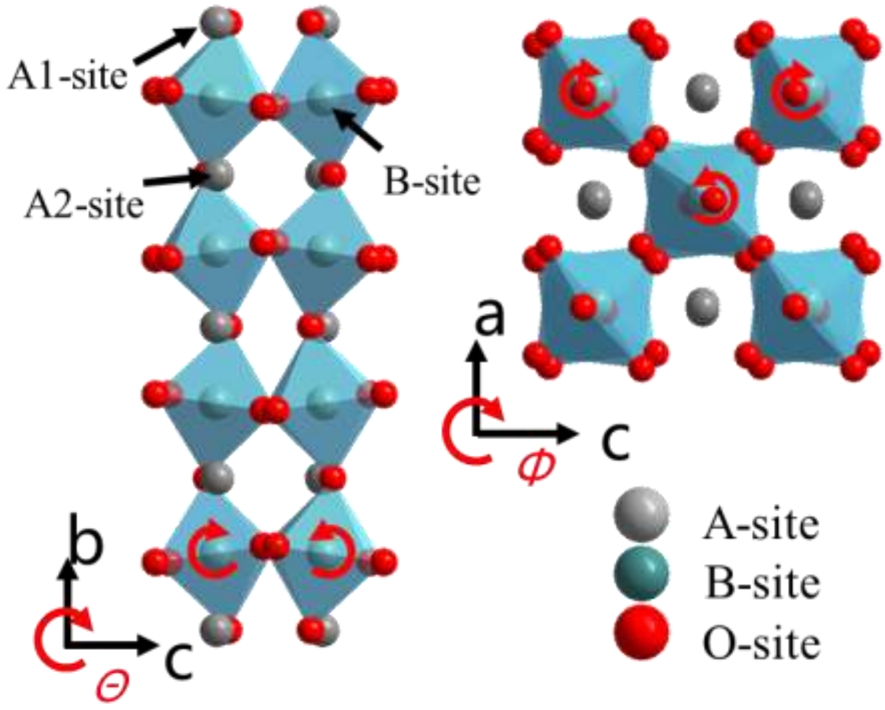

Supplementary Fig. 4 Schematic crystal structures and $[BO_6]$ octahedral tilting angles (Θ and Φ) of the Pbma space group.

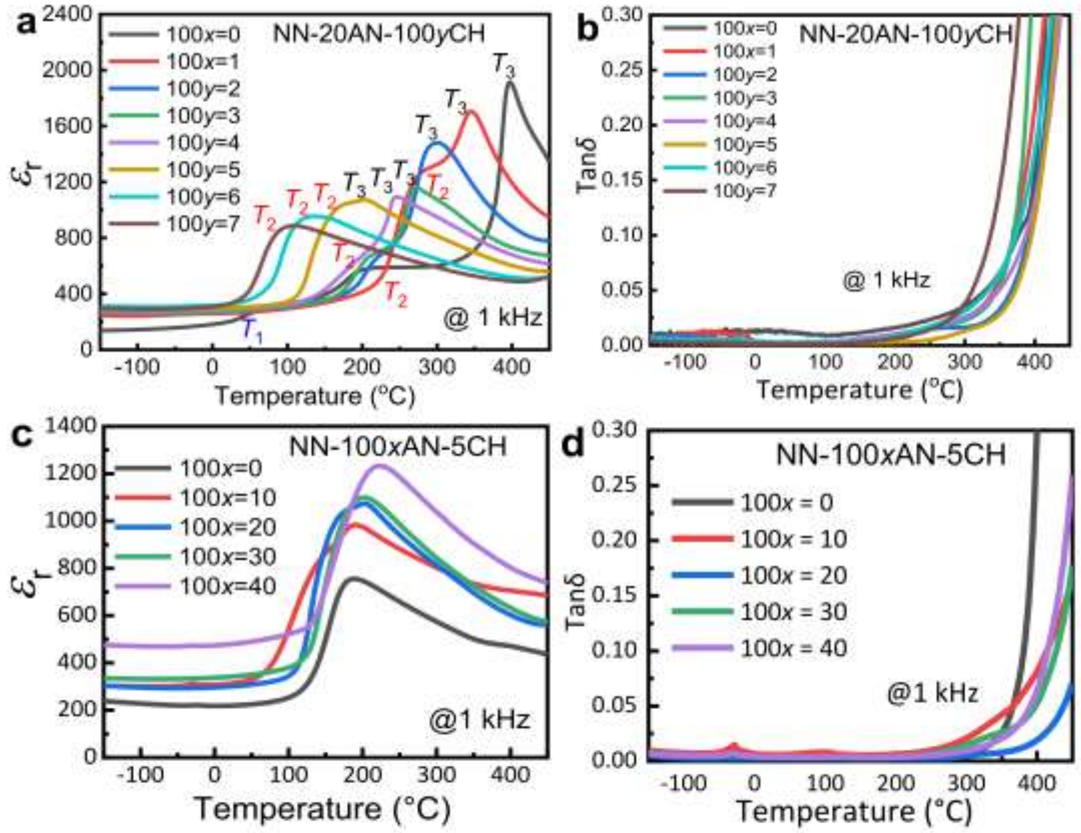

Supplementary Fig. 5 Temperature dependence of dielectric permittivity (ε_r) and loss (Tanδ) for a) ,b) NN-20AN-100*y*CH and c) ,d) NN-100*x*AN-5CH.

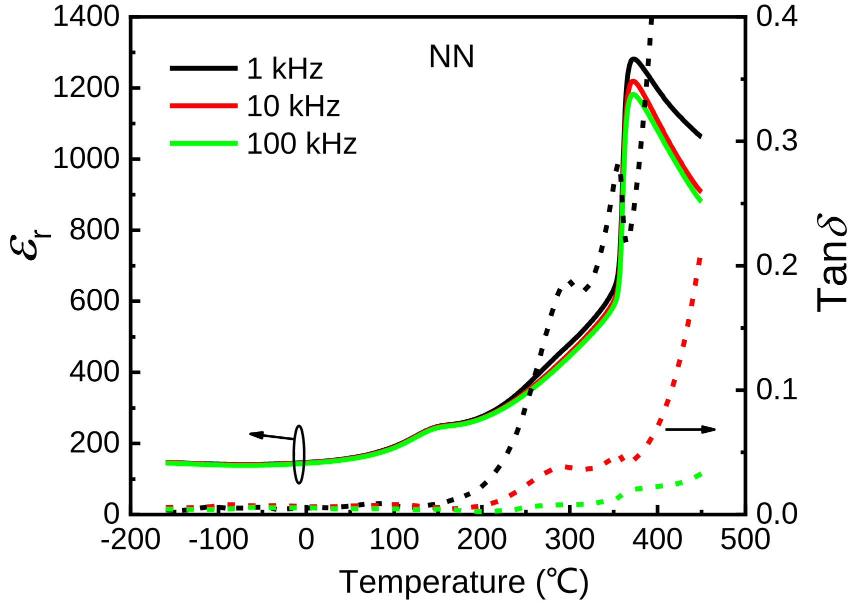

Supplementary Fig. 6 Temperature dependence of dielectric permittivity and loss for $NaNbO_3$.

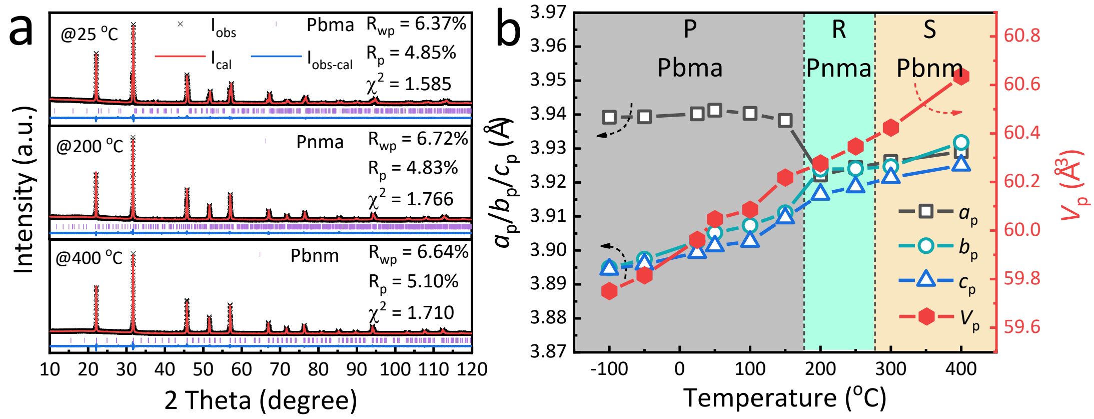

Supplementary Fig. 7 a) Rietveld refinement of XRD at some representative temperatures, and b) the resulted lattice parameters and cell volume at different temperatures for NN-20AN-5CH ceramics. All parameters are depicted in simple lattice.

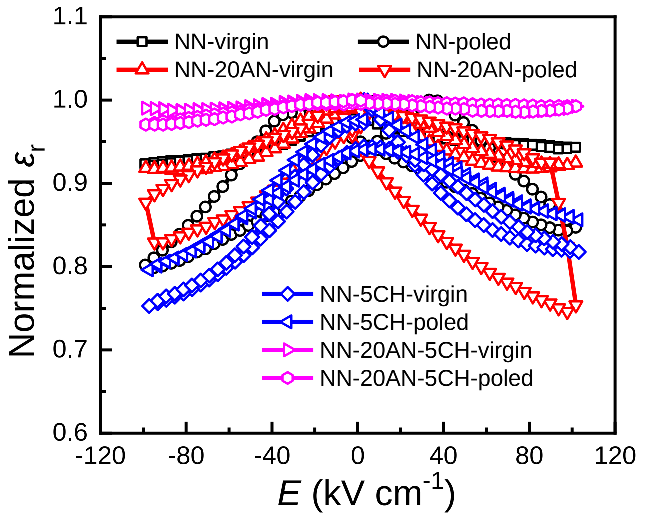

Supplementary Fig. 8 The ε_r-E curves under a maximum electric field of 100 kV cm^{-1} for the selected NN, NN-20AN, NN-5CH and NN-20AN-5CH ceramics at virgin and poled state.

Both virgin NN and AN exhibits moderate variations (~8%) in ε_r with increasing electric field, which increase obviously to ~20% for the poled ones due to the irreversible field-induced FE phase. The virgin and poled NN-5CH demonstrate large maximum variations in ε_r in the range of 20-25%, but the shape of ε_r-E curve is very similar. From the XRD analysis and *P*-*E* loop, the NN-5CH possesses an AFE P phase and good reversibility, which should stay at an AFE state before and after poling. In principle, it should exhibits small variations in ε_r. The abnormal change in ε_r may be associated with the low AFE-FE phase transition electric field (E_{AF}). The high applied electric field may induce gradual AFE-FE phase transition, thus leading to a very big change in ε_r. It's interesting that the NN-20AN-5CH exhibits electric field insensitive ε_r and the ε_r-E curves almost overlap at both virgin and poled states, indicating highly stable AFE feature.

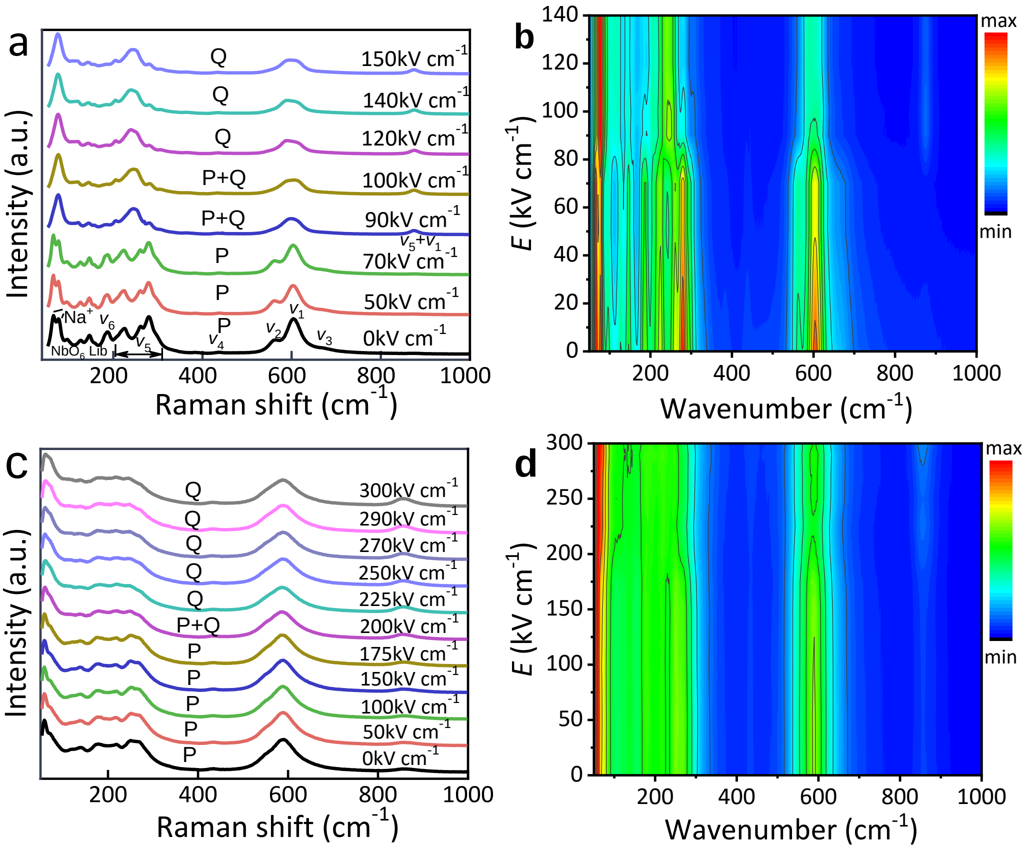

Supplementary Fig. 9 In-situ electric field dependence of Raman spectra for a), b) NN, and c), d) NN-20AN-5CH ceramics.

The NN exhibits typical Raman spectra that is generally observed in AFE P phase[4]. Partially overlapping Raman peaks associated with the Nb-O bonds are also observed in NN-20AN-5CH, due to the smaller difference between Nb-O bond lengths which is attributed to the decreased Nb displacement[3]. Whatsoever it still maintains the characteristics of P phase, similar to that of NN measured at high temperature[5, 6]. Low electric field has minimal impact on the Raman peaks of NN, while obvious changes can be observed at elevated electric fields. These changes include: 1) the lowest-lying bands at 66 and 77 cm^{-1} assigned to the translational modes of the two types of Na^+ ions in P phase gradually overlap with a broad peak centered around 75 cm^{-1} at 140 kV cm^{-1}; 2) the complex peaks associated with the internal vibrations (v_5, v_3, v_2, v_1) of the $[NbO_6]$ octahedra are weaker and overlap; 3) the intensity of the weak peak locating around 870.6 cm^{-1} (v_5+ v_1) enhances, similar to that observed in temperature and grain size induced FE phase in NN[4-6]. All these phenomena conform to an electric field induced P-to-Q phase transition, in consistence with the *P-E* measurement. Similar phenomenon can also be observed in NN-20AN-5CH, with an increased P-to-Q phase transition electric field over 200 kV cm^{-1}.

Supplementary Table 1 Rietveld-refinement results of SXRD by using the Rietveld method for some representative NN-based ceramics.

Sample	Space group	Lattice parameters				Displacement (Å)				Tilting angle (°)		R_{wp} (%)	R_p (%)	χ^2
		a (Å)	*b* (Å)	*c* (Å)	*V* (Å^3)	A1-site	A2-site	△A-site*	B-site	*Φ*	*Θ*			
NN	Pbma	5.5690	15.5267	5.5051	476.015	0.0699	0.1610	0.1155	0.0904	7.2377	10.4289	2.56	1.88	1.457
NN-5CH	Pbma	5.5611	15.5735	5.5021	476.511	0.0228	0.1218	0.0723	0.0834	6.1187	8.9529	3.80	2.64	3.032
NN-20AN-5CH	Pbma	5.5725	15.5488	5.5085	477.280	0.0236	0.1290	0.0763	0.0869	6.0575	8.8036	4.30	2.97	3.163

* △A-site is the average displacement of A1 and A2-site ions.

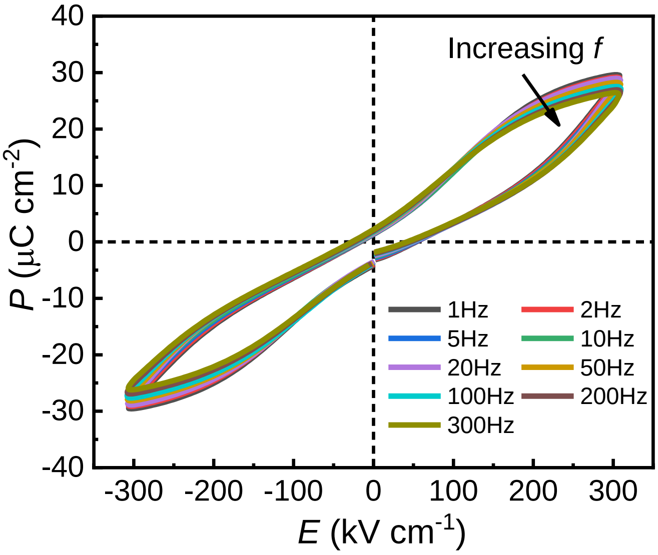

Supplementary Fig. 10 Frequency dependence of *P*-*E* loops for NN-20AN-5CH ceramic.

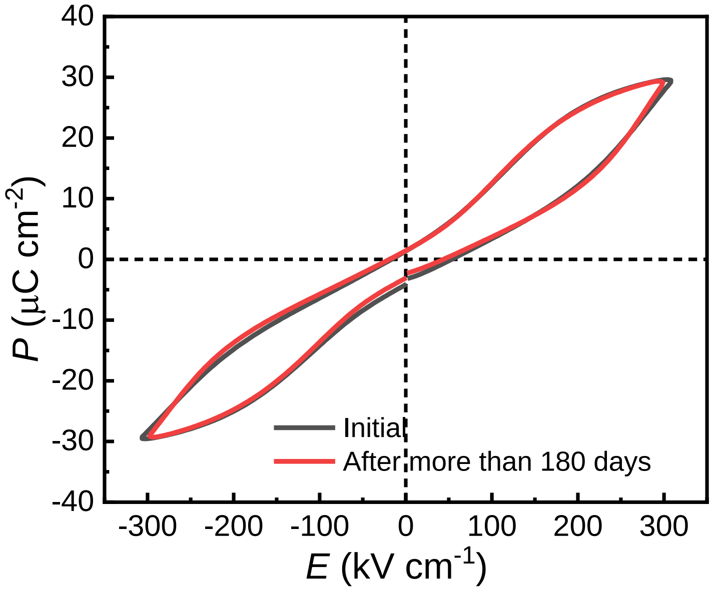

Supplementary Fig. 11 Aging time dependence of *P*-*E* loops for NN-20AN-5CH ceramic.

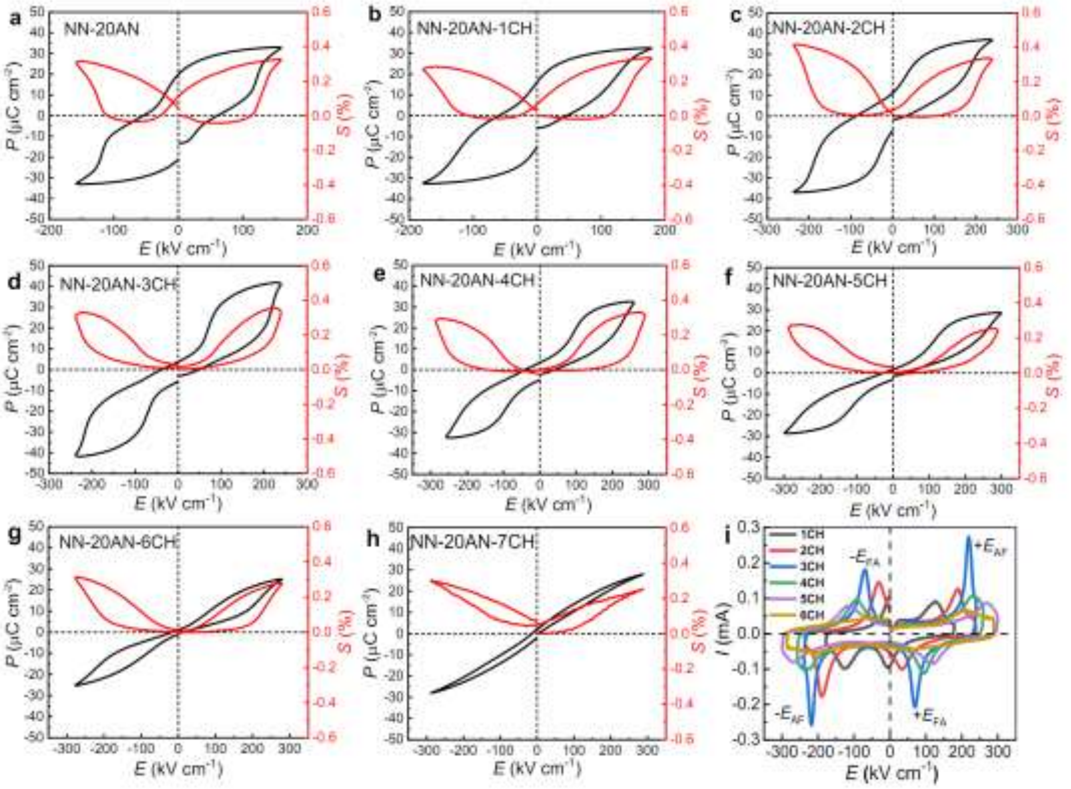

Supplementary Fig. 12 Effect of CH on the polarization, strain and current of NN-20AN system. *P*-*E* loops and *S*-*E* curves of a) NN-20AN, b) NN-20AN-1CH, c) NN-20AN-2CH, d) NN-20AN-3CH, e) NN-20AN-4CH, f) NN-20AN-5CH, g) NN-20AN-6CH, h) NN-20AN-7CH. i) *I*-*E* curves of all compositions.

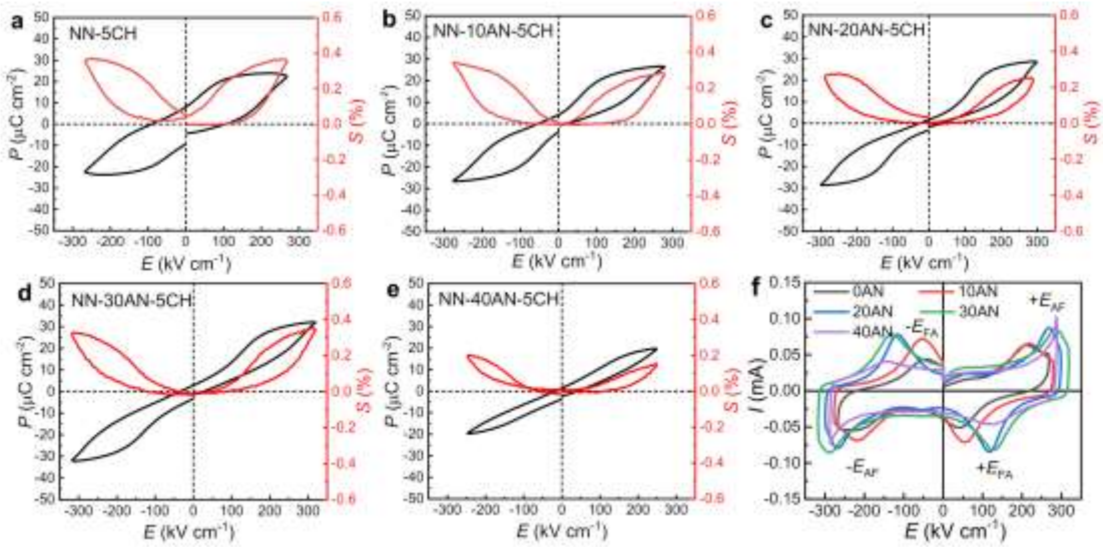

Supplementary Fig. 13 Effect of AN on the polarization, strain and current of NN-5CH system. *P*-*E* loops and *S*-*E* curves of a) NN-5CH, b) NN-10AN-5CH, c) NN-20AN-5CH, d) NN-30AN-5CH, e) NN-40AN-5CH. f) *I*-*E* curves of all compositions.

Supplementary Table 2 Electrical properties and stain of NN-100*x*AN-100*y*CH ceramics.

Composition	P_m (μC cm^{-2})	P_r (μC cm^{-2})	E_{AF} (kV cm^{-1})	E_{FA} (kV cm^{-1})	S_m (%)	S_{neg} (%)	ε_r	*Tan*δ	T_c (°C)
NN	27.9	24.9	-	-	0.092	-0.018	151	0.0051	373
NN-20AN	32.8	21.7	124	-25	0.326	-0.042	204	0.0125	397
NN-20AN-1CH	32.5	15.4	126	-7	0.334	-0.019	262	0.0021	346
NN-20AN-2CH	36.9	9.3	187	31	0.416	-0.006	279	0.0051	300
NN-20AN-3CH	41.2	4.7	219	69	0.355	0	274	0.0019	272
NN-20AN-4CH	32.1	3.9	229	95	0.331	-0.004	292	0.0028	246
NN-20AN-5CH	29.0	1.7	267	101	0.276	0	301	0.0022	203
NN-20AN-6CH	25.2	1.1	222	77	0.309	0	328	0.0026	136
NN-20AN-7CH	28.0	1.9	-	-	0.300	0	334	0.0012	108
NN-5CH	22.4	8.8	208	40	0.369	-0.007	220	0.0026	189
NN-10AN-5CH	26.5	4.0	220	53	0.339	-0.004	314	0.0052	190
NN-30AN-5CH	31.9	3.0	286	127	0.351	-0.020	416	0.0021	203
NN-40AN-5CH	24.3	2.3	287	128	0.203	-0.007	479	0.0028	223

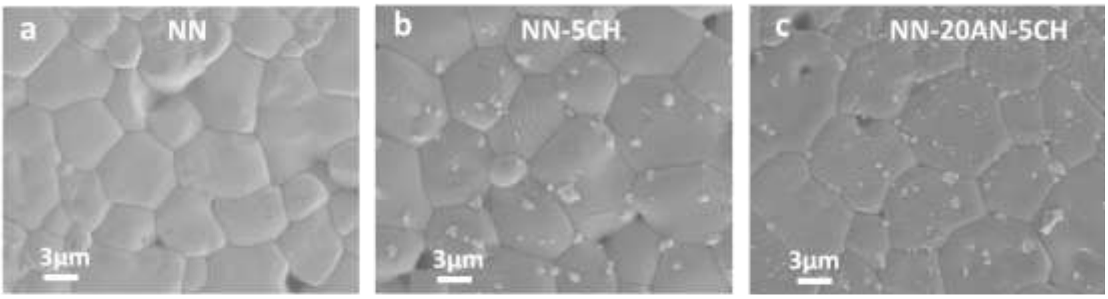

Supplementary Fig. 14 SEM images of a) NN, b) NN-5CH, and c) NN-20AN-5CH ceramics.

All samples show equiaxed grains with closely compacted microstructure, except a small number of small particles (Hf-rich oxide) on the surface of matrix grains in CH modified NN-based ceramics. It should be noted the amount of impurity can be neglected, because no detectable extra peak is observed in the XRD. The size distribution of most grains is around 5-10μm, indicating the AN and CH modifications do not significantly change the grain size and morphology. All these results support the strong AFE features in NN-20AN-5CH ceramic are not caused by the changes in grain size and morphology.

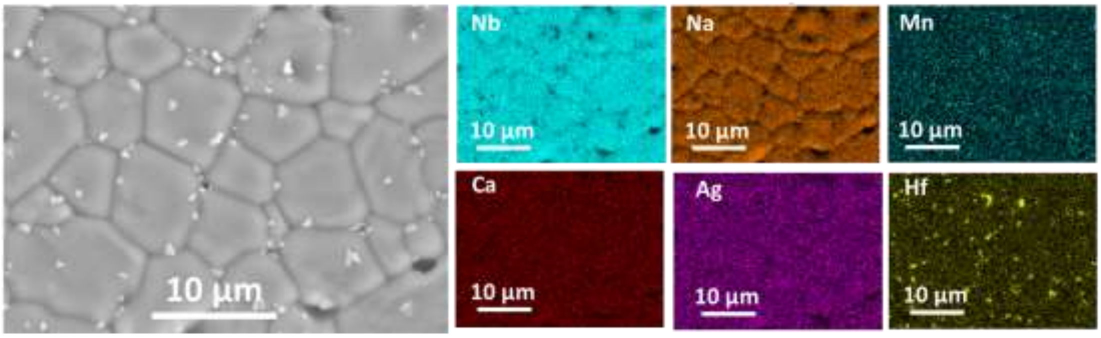

Supplementary Fig. 15 SEM and elemental mapping images of NN-20AN-5CH ceramic.

Some small particles with white contrast were observed on the surface of the matrix grains. From the EDS, it can be found that Nb, Na, Mn, Ag and Ca are uniformly distributed in the samples. Most of the Hf element is also uniformly distributed in the samples, but with small amount of Hf-rich oxide aggregates in the form of small particles with size of 200-500 nm on the surface.

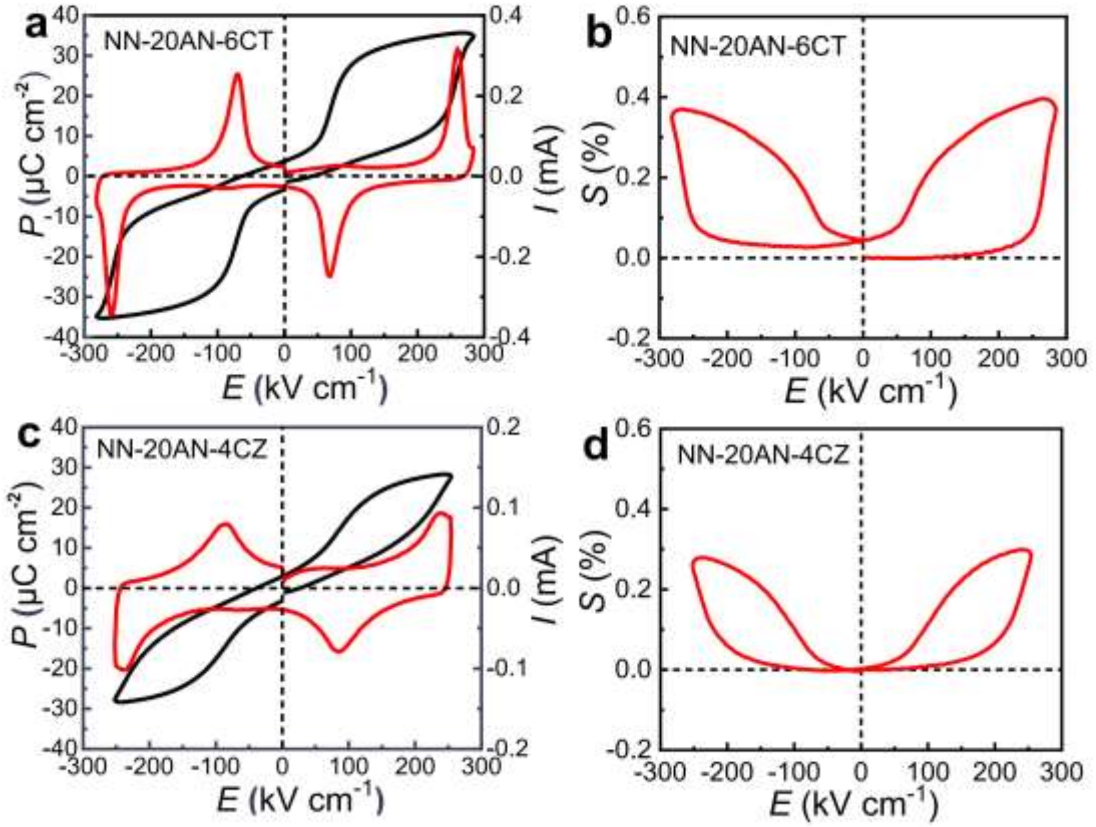

Supplementary Fig. 16 The polarization, strain and current of some newly developed NN-based antiferroelectrics. a) *P*-*E* loops, *I*-*E* curves, and b) *S*-*E* curves of NN-20AN-6CT. c) *P*-*E* loops, *I*-*E* curves, and d) *S*-*E* curves of NN-20AN-4CZ.

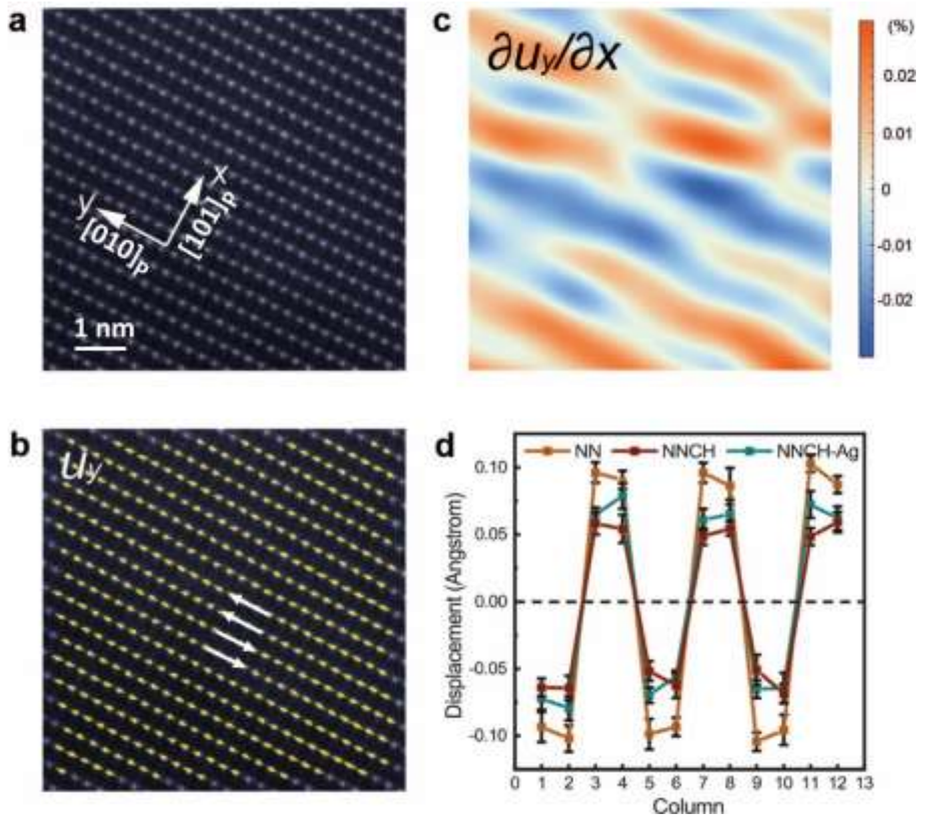

Supplementary Fig. 17 a) ADF image for NN-5CH. b) B-site displacement map for NN-5CH. (c) Displacement derivative $\frac{\partial u_y}{\partial x}$ from an indicating displacement wave of NN using geometric phase analysis (GPA). (d) Averaged B-site displacement profiles of NN, NN-5CH and NN-20AN-5CH.

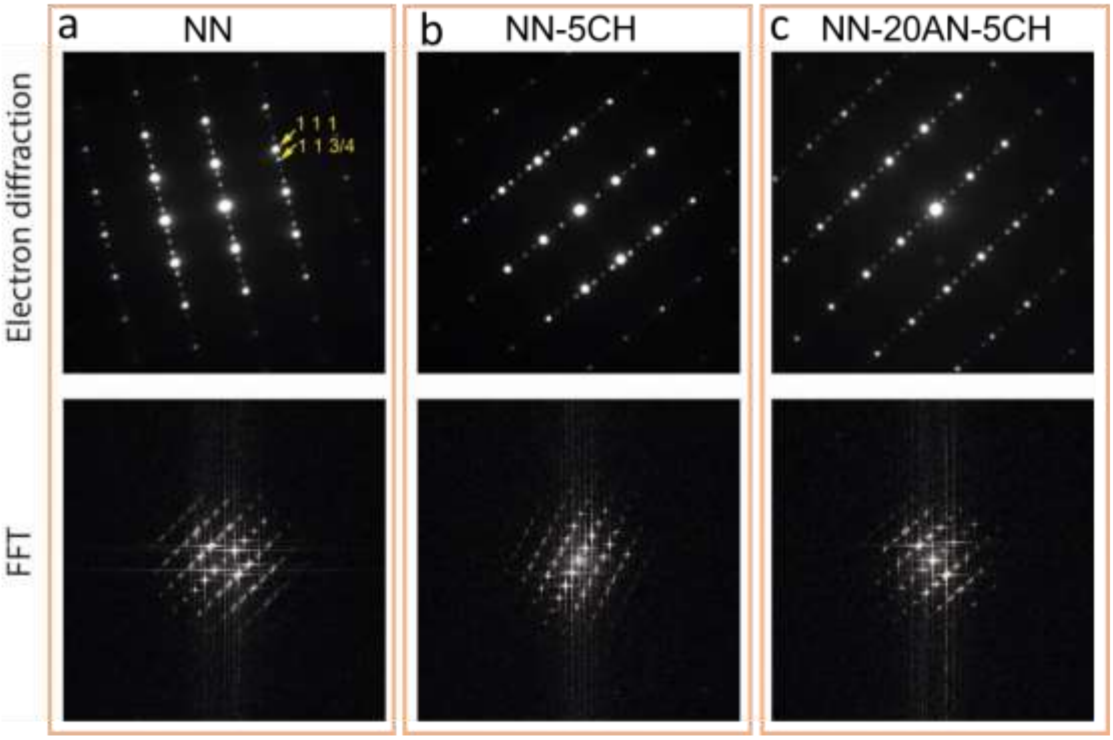

Supplementary Fig. 18 Electron diffractions for a) NN, b) NN-5CH and c) NN-20AN-5CH taken on $[10\bar{1}]_p$ zone axis and the FFT images of Fig. 5a, Supplementary Fig. 17 and Fig. 5b, respectively.

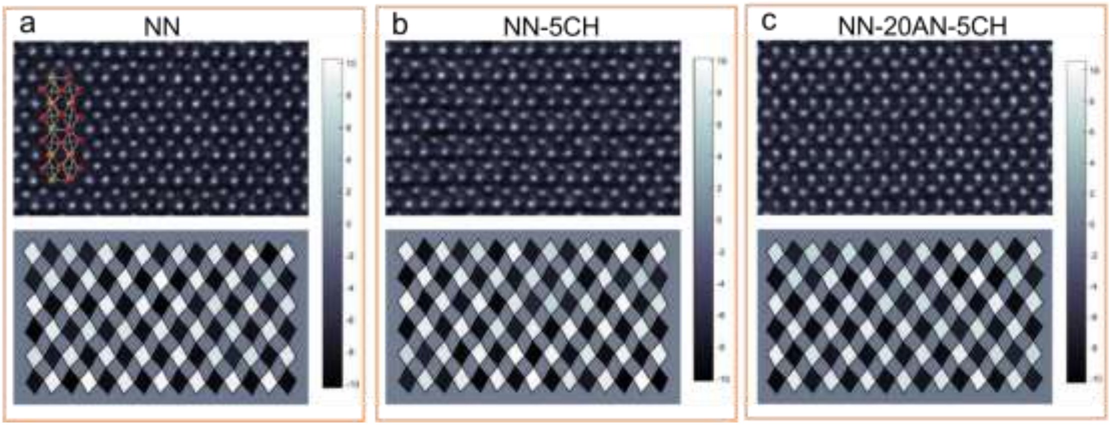

Supplementary Fig. 19 Bright field images and maps of the antiphase oxygen octahedral tilt around $[101]_p$ axis for a) NN, b) NN-5CH and c) NN-20AN-5CH.

Supplementary References

1 Htet CS*, et al.* Atomic structural mechanism for ferroelectric-antiferroelectric transformation in perovskite $NaNbO_3$. *Phys Rev B* **105**, 174113 (2022).

2 Shimizu H, Guo H, Reyes-Lillo SE, Mizuno Y, Rabe KM, Randall CA. Lead-free antiferroelectric: $xCaZrO_3$-(1-x)$NaNbO_3$ system (0≤x≤0.10). *Dalton Transactions* **44**, 10763-10772 (2015).

3 Qi H, Xie A, Fu J, Zuo R. Emerging antiferroelectric phases with fascinating dielectric, polarization and strain response in $NaNbO_3$-$(Bi_{0.5}Na_{0.5})TiO_3$ lead-free binary system. *Acta Mater* **208**, 116710 (2021).

4 Shiratori Y, Magrez A, Dornseiffer J, Haegel FH, Pithan C, Waser R. Polymorphism in micro-, submicro-, and nanocrystalline $NaNbO_3$. *J Phys Chem B* **109**, 20122-20130 (2005).

5 Shakhovoy RA*, et al.* Ferroelectric Q and antiferroelectric P phases' coexistence and local phase transitions in oxygen-deficient $NaNbO_3$ single crystal: micro-Raman, dielectric and dilatometric studies. *J Raman Spectrosc* **43**, 1141-1145 (2012).

6 Lima R*, et al.* Temperature-dependent Raman scattering studies in $NaNbO_3$ ceramics. *J Raman Spectrosc* **385**, 1-6 (2002).
